# Conservative versus surgical management for patients with rotator cuff tears: a systematic review and META-analysis

**DOI:** 10.1186/s12891-020-03872-4

**Published:** 2021-01-08

**Authors:** Umile Giuseppe Longo, Laura Risi Ambrogioni, Vincenzo Candela, Alessandra Berton, Arianna Carnevale, Emiliano Schena, Vincenzo Denaro

**Affiliations:** 1 Department of Orthopaedics and Trauma Surgery, Campus Bio-Medico University of Rome, Via Alvaro del Portillo, 200, 00128 Trigoria, Rome, Italy; 2 Unit of Measurements and Biomedical Instrumentation, Campus Bio-Medico University of Rome, Via Alvaro del Portillo, 200, 00128 Trigoria, Rome, Italy

**Keywords:** Surgery, Surgical treatment, Conservative treatment, Physiotherapy, Rotator cuff, Rotator cuff tear, Rotator cuff repair, Shoulder, Pain

## Abstract

**Background:**

This study aims to compare conservative versus surgical management for patients with full-thickness RC tear in terms of clinical and structural outcomes at 1 and 2 years of follow-up.

**Methods:**

A comprehensive search of CENTRAL, MEDLINE, EMBASE, CINAHL, Google Scholar and reference lists of retrieved articles was performed since the inception of each database until August 2020. According to the Cochrane Handbook for Systematic Reviews of Interventions, two independent authors screened all suitable studies for the inclusion, extracted data and assessed risk of bias. Only randomised controlled trials comparing conservative and surgical management of full-thickness RC tear in adults were included. The primary outcome measure was the effectiveness of each treatment in terms of Constant-Murley score (CMS) and VAS pain score at different time points. The secondary outcome was the integrity of the repaired tendon evaluated on postoperative MRI at different time points. The GRADE guidelines were used to assess the critical appraisal status and quality of evidence.

**Results:**

A total of six articles met the inclusion criteria. The average value of CMS score at 12 months of follow-up was 77.6 ± 14.4 in the surgery group and 72.8 ± 16.5 in the conservative group, without statistically significant differences between the groups. Similar results were demonstrated at 24 months of follow-up. The mean of VAS pain score at 12 months of follow-up was 1.4 ± 1.6 in the surgery group and 2.4 ± 1.9 in the conservative group. Quantitative synthesis showed better results in favour of the surgical group in terms of VAS pain score one year after surgery (− 1.08, 95% CI − 1.58 to − 0.58; *P* < 0.001).

**Conclusions:**

At a 2-year follow-up, shoulder function evaluated in terms of CMS was not significantly improved. Further high-quality level-I randomised controlled trials at longer term follow-up are needed to evaluate whether surgical and conservative treatment provide comparable long-term results.

## Background

Shoulder pathologies are incrementing at a rapid rate [1]. Every year in the United States, 4.5 million medical visits are made for shoulder diseases, of which 70% is mainly due to rotator cuff (RC) tears [1]. Even though RC tears may be asymptomatic, some patients complain symptoms ranging from minimal discomfort to severe joint pain, muscle weakness and marked dysfunction with significant limitation in the activities of daily living [2]. Approximately 65% of RC repairs are performed annually in patients ageing < 65 years, thus profoundly affecting the working population [3–10].

RC tears are classified in partial or full-thickness tears according to the severity of the tendon fibres disruption and the communication between the subacromial and glenohumeral space [9, 11]. The management of RC tears is a relevant topic with a wide prevalence, but what is the optimal treatment for partial and full-thickness RC tears is still unclear since both conservative and surgical treatment have strengths and weaknesses [12–17]. Despite the high numbers of procedures performed all over the world, structural failures of RC surgery are very high, ranging from 16 to 94% [18, 19]. Moreover, it is not clear whether reattaching the tendon to the bone can avoid the progression of muscle atrophy and degeneration [18]. The surgical treatment of RC tears is a well-documented therapeutic option for youngers with acute symptomatic partial and full-thickness tears and severe dysfunction [12, 20–22]. In contrast, the conservative treatment is widely used in patients with a degenerative condition of the tendons or tendon disruption of less than 50% of the entire tendon thickness [23–26]. On the contrary, conservative treatment may predispose patients to continued irreversible tissue degeneration over time. Therefore, RC tears which initially could be managed as repairable may become irreparable, leading to the need for further treatments and worse results [27]. The duration of non-operative management is one of the most challenging factors of the conservative approach. This type of treatment should be adapted to the patient’s characteristics in terms of clinical and structural outcomes, lifestyle, level of functional impairment, and compliance with the physical sessions [28]. However, also the surgical treatment depends on the size of the tear, the quality of the tendon, the staging of the retraction, the tear pattern and the experience of the surgeon [29]. This suggests that several structural and contextual factors can contribute to the success of both conservative and surgical treatment, justifying the considerable heterogeneity and complexity of comparison [27].

To date, only a few randomised controlled trials have been performed to compare the effects of conservative and surgical management. Some of these randomised controlled trials provided results at short-term follow-up (≤1 year) [12, 15, 30]. In contrast, others provided results at two years after the intervention [13, 14, 31], and only one provided results at 5 and 10 years of follow-up [13, 31]. In previous meta-analyses, short-term follow-up outcomes for both conservative and surgical RC treatment were compared [32–34]. Results showed limited evidence about the superiority of the surgical treatment over the conservative one, stating the need for studies with medium-term follow-up (1 < years ≤3) and long-term follow-up (years > 3). To date, to the best of our knowledge, meta-analyses at longer term follow-up are not available.

Therefore, this study aimed to compare conservative versus surgical management for patients with full-thickness RC tear in terms of clinical and structural outcomes.

## Methods

### Search strategy and study selection

According to the PRISMA (Preferred Reporting Items for Systematic Reviews and Meta-analyses) checklist and algorithm [35], a comprehensive search of the Cochrane Register of Controlled Trials (CENTRAL), MEDLINE (Ovid), EMBASE (Ovid), CINAHL (EBSCO), Google Scholar and reference lists of retrieved articles was performed. The combination of free-text terms and Medical Subject Headings (MeSH) in title and abstract was used to perform the research. The search strategy was built on the application of Boolean logic operators to the following keywords: *(“rotator cuff” OR “rotator cuff tear” OR “rotator cuff injury” OR “non-traumatic tears” OR “rotator cuff rupture” OR “rotator cuff disease”) AND (“rotator cuff repair” OR “surgical procedures” OR “rotator cuff surgery” OR “arthroscopy” OR “operative” OR “non operative” OR “conservative” OR “treatment” OR “management”)*. After duplicates removal, two independent reviewers (L.R.A. and V.C) had verified the suitability of each article published in a peer-reviewed journal for the relevance of title and abstract to the objective of this study without excluding any journal since the inception of each database until August 2020. Studies without abstract or meaningful information were excluded during the study selection process. The independent reviewers conducted an accurate full-text reading of the chosen articles, obtaining data to reduce selection bias. Due to the language skills of the authors, articles in English, French, Spanish, German, and Italian were investigated. Any disagreement among investigators on the inclusion of a study was resolved by the senior investigator (V.D.), who made the final decision. Finally, to avoid potential biases, the selected articles, the list of references and the articles excluded from the study were reviewed, evaluated and discussed by all the authors.

Articles were included whether they fulfilled the following inclusion criteria: randomised controlled trial, full-thickness rotator cuff tear, and age ≥ 18. Furthermore, to increase the strength of the study, only level-I studies based on the Oxford Centre of EBM published in peer-reviewed journals were included [36]. Articles were considered ineligible for this study if one of the following exclusion criteria was present: a follow-up period of less than one-year, previous shoulder surgery. Although some contextual and structural factors (e.g. partial or complete rupture, age of tearing, degenerative nature of the disease) may have been important to consider in the eligibility criteria, the heterogeneity of participants in the included studies forced us to broaden the inclusion criteria.

### Data extraction

Extraction data was performed by the two reviewers (U.G.L and L.R.A) using a predetermined form to ensure consistency of appraisal. For each article included in the study, the following data has been extracted: authors, year, study design, level of evidence, sample size, losses at follow-up, number of patients in the surgical and conservative group, sex, age, follow-up, clinical outcomes (Constant-Murley score (CMS), Pain-free abduction, Range of Motions (ROMs), Simple Shoulder Test (SST) score, American Shoulder and elbow surgeons (ASES) score), visual analog scale (VAS) score, retear events and adverse effects.

Our primary outcome measure was the effectiveness of each treatment in terms of clinical outcome at different time points (CMS and VAS pain score). The secondary outcome was the integrity of the repaired tendon evaluated on postoperative MRI at different time points. There were no reported adverse effects.

### Data synthesis and statistical analysis

Continuous variables were reported as mean ± standard deviation (SD) with 95% confidence intervals (CI). Dichotomous data were reported as risk ratio (RR) with 95% CI. In all studies, *P*-value < 0.05 was considered statistically significant. Whether at least two studies compared the same variables, a random or a fixed effect based on heterogeneity was calculated in a meta-analysis. Review Manager (RevMan, version 5 for Windows; Cochrane Information Management System) was used to perform the meta-analysis. The results of the individual studies and meta-analysis are presented with the forest plots.

### Assessment of heterogeneity

The assessment of heterogeneity was accomplished through the visual examination of forest plots and overlapping CIs, and by I^2^ statistics.

The assessment of the clinical and methodological characteristics of the included studies (e.g. differences in participants, fairness in the number of participants among intervention groups, interventions, losses at follow-up, clinical outcome evaluations) was used to explore the clinical heterogeneity. When clinical heterogeneity was assessed as low, we pooled the data in the meta-analysis. Otherwise, we discussed whether to exclude some studies altogether or include them after a sensitivity analysis.

Between-studies heterogeneity was evaluated in terms of *I*^2^ index. We considered a *P* value of less than 0.10 as evidence of heterogeneity. According to the Cochrane Handbook for Systematic Reviews of Interventions, the interpretation of the *I*^2^ for heterogeneity was as follows:

- 0 to 40%, was not important
- 30 to 60%, represented moderate heterogeneity
- 50 to 90%, represented substantial heterogeneity
- 75 to 100%, represented considerable heterogeneity

A fixed-effect model in the data synthesis was adopted when heterogeneity values were ≤ 60%; otherwise, a random-effects model was used.

### Risk of bias

Two independent reviewers (L.R.A and U.G.L.) assessed the risk of bias for each included study. The Cochrane Risk of Bias has been used as a tool for critical appraisal. Following methods recommended by The Cochrane Collaboration, a domain-based evaluation (random sequence generation; allocation concealment; blinding of participants, personnel and outcome assessors; incomplete outcome data; selective outcome data reporting and other sources of bias) was performed [37]. The following judgments were used: low risk, high risk, or unclear (either lack of information or uncertainty over the potential for bias). Authors resolved disagreements by consensus, and a third author (V.D.) was consulted to resolve disagreements whether necessary.

### Quality assessment

The GRADE (Grading of Recommendations Assessment, Development and Evaluation) guidelines were used to assess the critical appraisal status and quality of evidence of the included randomised controlled trials. The combination of four factors (i.e., study design, study quality, consistency, and directness) provided whether the quality of the evidence was high, moderate, low, or very low. We downgraded the evidence quality from’ high quality’ by one level for serious risk of bias, inconsistency, indirectness of evidence, imprecision of effect estimates or potential publication bias. The following outcomes were included in the’ Summary of findings’ tables:

- CMS at one year of follow-up
- CMS at two years of follow-up
- VAS pain score at one year of follow-up

## Results

### Search results and data extraction

**Fig. 1:**
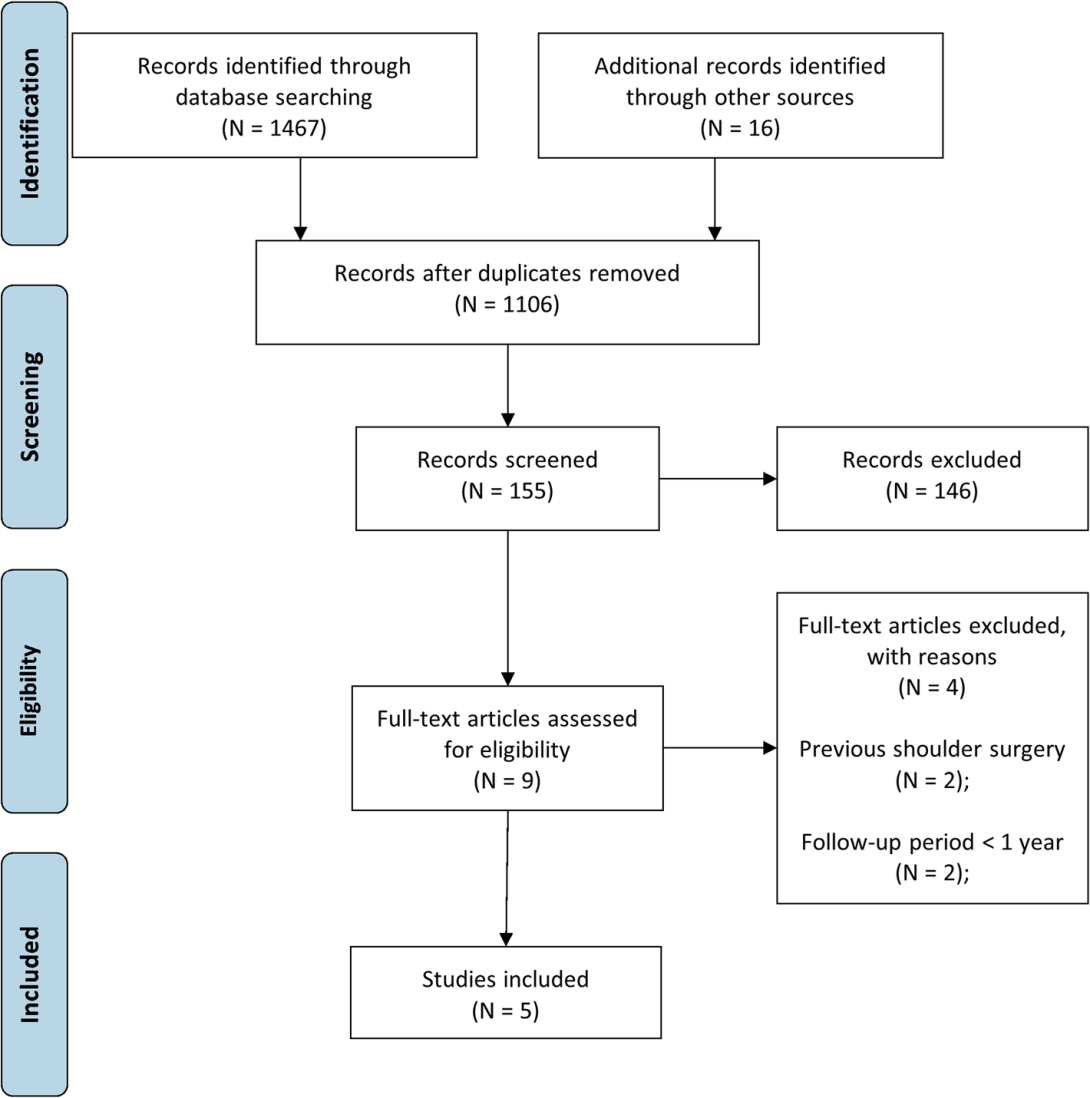
PRISMA 2009 flow diagram

The search strategy yielded a total of 1467 articles, to which 16 articles were added from the reference list of included studies. After duplicates removing, 1106 articles remained for review. A total of 951 articles were excluded because they did not report specific data on the management of RC tears. The remaining 155 full-text articles were evaluated; of these, only nine articles were potentially eligible. Of these, only six articles met the inclusion criteria [12–15, 30, 31]. 3 of 6 performed by Moosmayer et al. [12, 13, 31], and 2 of 6 [14, 30] performed by Kukkonen et al. are publications of results from the same study group at different follow-up times. For this reason, six studies were included in the meta-analysis, but the patient cohorts from which the data were extracted are three (Fig. 1). The absence of further long term follow-up studies precluded the possibility to compare data from the only available 10-year follow-up randomised controlled trial conducted by Moosmayer et al. [13]. Kukkonen et al. included a cohort of patients who underwent physical therapy and subacromial decompression without RC repair [14, 30]. Therefore, we excluded data related to this study group, as they did not meet the inclusion criteria. Patients were evaluated at different follow-up periods. In particular, clinical outcomes were reported at 12 months in 3 studies [12, 15, 30], at 24 months in 2 studies [14, 31], at 5 and 10 years in 1 study [13]. MRI was used to report structural outcomes at one year of follow-up in 2 studies [12, 15] and 24 months in 1 study [14]. Ultrasound was used to report structural outcomes at 5 and 10 years in 1 study [13]. Therefore, comparison for clinical outcomes was possible at 1 and 2 years of follow-up and comparison for structural outcome was possible at one year of follow-up. Further study characteristics are summarised in Table 1.

**Table 1:** Demographics

AUTHORS	GEOGRAPHIC AREA	No OF INVOLVED CENTERS	PARTICIPANTS			AGE	SEX	FOLLOW-UP	CLINICAL OUTCOMES	STRUCTURAL OUTCOMES
			At baseline	Lost at follow-up	At maximum follow-up					
*Kukkonen* et al*, 2014* [29]	Europe	1	180	13	167	SG = 65 CG = 65	SG = 26 M, 29 F CG = 24 M; 31 F	12 months	CMS	–
*Kukkonen* et al.*, 2015* [14]							SG = 29 M, 25 F CG = 22 M; 33 F	24 months	CMS, VAS	MRI at 24 months
*Lambers****-****Heerspink* et al.*, 2015* [15]	Europe	1	56	11	45	SG = 61 CG = 61	SG = 15 M; 10 F CG = 20 M; 11 F	12 months	CMS, VAS	MRI at 12 months
*Moosmayer* et al*, 2010* [12]	Europe	1	103	10	93	SG = 59 CG = 61	SG: 37 M; 15 F CG: 36 M; 15 F	12 months	CMS, ASES, VAS	MRI at 12 months
*Moosmayer* et al.*, 2014* [30]				10	93			24 and 60 months		MRI at 12 months and Ultrasound at 60 months
*Moosmayer* et al.*, 2019* [13]				12	91			120 months		MRI at 12 months and Ultrasound at 60 months and 120 months

*SG* Surgical group, *CG* Conservative group, *M* Male, *F* Female, *MRI* Magnetic Resonance Imaging, *CMS* Constant-Murley score, *VAS* Visual analogue scale, *ASES* American shoulder and elbow surgery

Study characteristics at different follow-up times are summarised in Table 2.

**Table 2:** Studies characteristics at different follow-up times

	Follow-up, months	Patients at 12 months of follow-up, n	Patients at 24 months of follow-up, n	Patients included in quantitative analysis at the maximum follow-up period, n	
				Conservative group	Surgical group
*Kukkonen* et al *2014,* [29]	3, 6, 12	110	–	55	55
*Kukkonen* et al *2015,* [14]	3, 6, 12, 24	–	109	55	54
*Lambers-Heerspink* et al *2015,* [15]	12	45	–	25	20
*Moosmayer* et al *2010,* [12]	6, 12	102	–	51	51
*Moosmayer* et al *2014,* [30]	6, 12, 24, 60	–	101	51	51

### Meta-analysis results

Meta-analysis was performed to investigate the potential differences between conservative and surgical management for patients with RC tears in terms of CMS (at 12 months and 24 months of follow-up) and VAS pain score (at 12 months of follow-up). Each study evaluated the shoulder function through several outcomes (e.g., American Society of Shoulder and Elbow Surgeon, Pain-free abduction, Dutch Simple Shoulder Test, Range of Motion). However, the comparison between all the included articles was possible only in terms of CMS and the VAS pain score.

#### CMS score at 12 months of follow-up

**Fig. 2:**
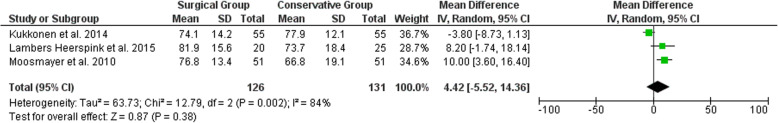
Forest plot: Constant and Murley score at 12 months of follow-up

The CMS score at 12 months of follow-up was recorded in 3 studies [12, 15, 30]. Data from 257 patients (126 in the surgical group and 131 in the conservative group) were presented in Table 3 and depicted graphically in Fig. 2. The average value at 12 months of follow-up was 77.6 ± 14.4 in the surgery group and 72.8 ± 16.5 in the conservative group. Results showed that there are no statistically significant differences between the CMS measured at one year of follow-up between patients undergoing surgical RC repair and patients treated conservatively (4.42, 95% CI − 5.52 to 14.36; *P* = 0.38, I^2^ = 84%). Because of the extensive heterogeneity of the cohort under examination, a random effect was used.

**Table 3:** Constant and Murley Score (mean ± SD) at baseline, 12 and 24 months of follow-up

**Constant and Murley score at 1-year follow-up (range 0 to 100)**						
**Authors**	***Moosmayer 2010,*** [12]		***Kukkonen 2014,*** [30]		***Lambers Heerspink 2015,*** [15]	
	**Surgical group** **(*n*** **= 51)**	**Conservative group** **(*n*** **= 51)**	**Surgical group** **(*n*** **= 55)**	**Conservative group** **(*n*** **= 55)**	**Surgical group** **(*n*** **= 20)**	**Conservative group** **(*n*** **= 25)**
**Baseline**	35.3 ± 13.2	38.4 ± 14.2	57.1 ± 16.7	58.1 ± 13.2	55.6 ± 18.4	56.9 ± 15.0
**12 months**	76.8 ± 13.4	66.8 ± 19,1	74.1 ± 14.2	77.9 ± 12.1	81.9 ± 15.6	73.7 ± 18.4
**Constant and Murley score at 2-year follow-up (range 0 to 100)**						
**Authors**	***Moosmayer 2014,*** [31]		***Kukkonen 2015,*** [14]		–	
	**Surgical group** **(*n*** **= 51)**	**Conservative group** **(*n*** **= 51)**	**Surgical group** **(*n*** **= 54)**	**Conservative group** **(*n*** **= 55)**		
**Baseline**	35.3 ± 13.2	38.4 ± 14.2	57.1 ± 16.7	58.1 ± 13.2		
**24 months**	79.3 ± 13.6	77.7 ± 14.9	76.2 ± 29.1	80.6 ± 29.9

#### CMS score at 24 months of follow-up

**Fig. 3:**
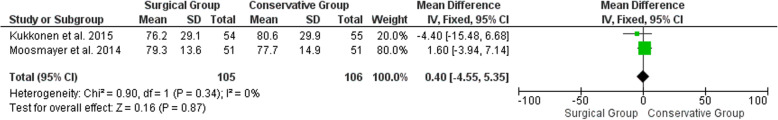
Forest plot: Constant and Murley score at 24 months of follow-up

The CMS score at 24 months of follow-up was recorded in 2 studies [14, 31]. 211 patients (105 in the surgical group, and 106 in the conservative group) were included (Table 3). The average value at 24 months follow-up was 77.9 ± 21.4 in the surgery group and 79.1 ± 22.4 in the conservative group. Results showed that there are no statistically significant differences between the CMS measured at two years of follow-up between patients undergoing surgical RC repair and patients treated conservatively (0.40, 95% CI − 4.55 to 5.35; *P* = 0.87, I^2^ = 0%) (Fig. 3). As opposed to the one-year CMS assessment, the homogeneity of the sample population allowed a fixed effect to be used.

#### VAS score at 12 months of follow-up

**Fig. 4:**
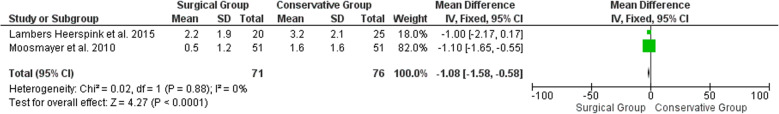
Forest plot: VAS pain score at 12 months of follow-up

The VAS pain score at 12 months of follow-up was recorded in 2 studies [12, 15]. 147 patients (71 in the surgical group, and 76 in the conservative group) were included (Table 4). The mean of VAS pain score was 1.4 ± 1.6 in the surgery group and 2.4 ± 1.9 in the conservative group. The surgery group provided superior results when compared to the conservative group in terms of VAS pain score at 12 months of follow-up (− 1.08, 95% CI − 1.58 to − 0.58; *P* < 0.001, I^2^ = 0%) (Fig. 4). The homogeneity of the sample population allowed a fixed effect to be used.

**Table 4:** VAS pain score (mean ± SD) at baseline and 12 months of follow-up

VAS pain score at 1-year follow-up (range 0 to 10)				
Authors	Moosmayer 2010, [12]		Lambers Heerspink 2015, [15]	
	Surgical group (***n*** = 51)	Conservative group (***n*** = 51)	Surgical group (***n*** = 20)	Conservative group (***n*** = 25)
**Baseline**	5.6 ± 2.0	5.3 ± 1.9	6.7 ± 1.7	6.3 ± 1.3
**12 months**	0.5 ± 1.2	1.6 ± 1.6	2.2 ± 1.9	3.2 ± 2.1

The VAS pain score at 24 months of follow-up was not performed because the VAS pain score ​​in one article has been reported only graphically, therefore it was not possible to accurately extract numerical data [14].

### Structural outcomes at 12 months of follow-up

MRI results were reported for the surgical group at 12 months of follow-up in 2 studies [12, 15]. 69 patients (50 and 19 respectively) were included. 24 (35%) retears were found (10 and 14 respectively) at a 1-year follow-up.

### Quality assessment results

**Fig. 5:**
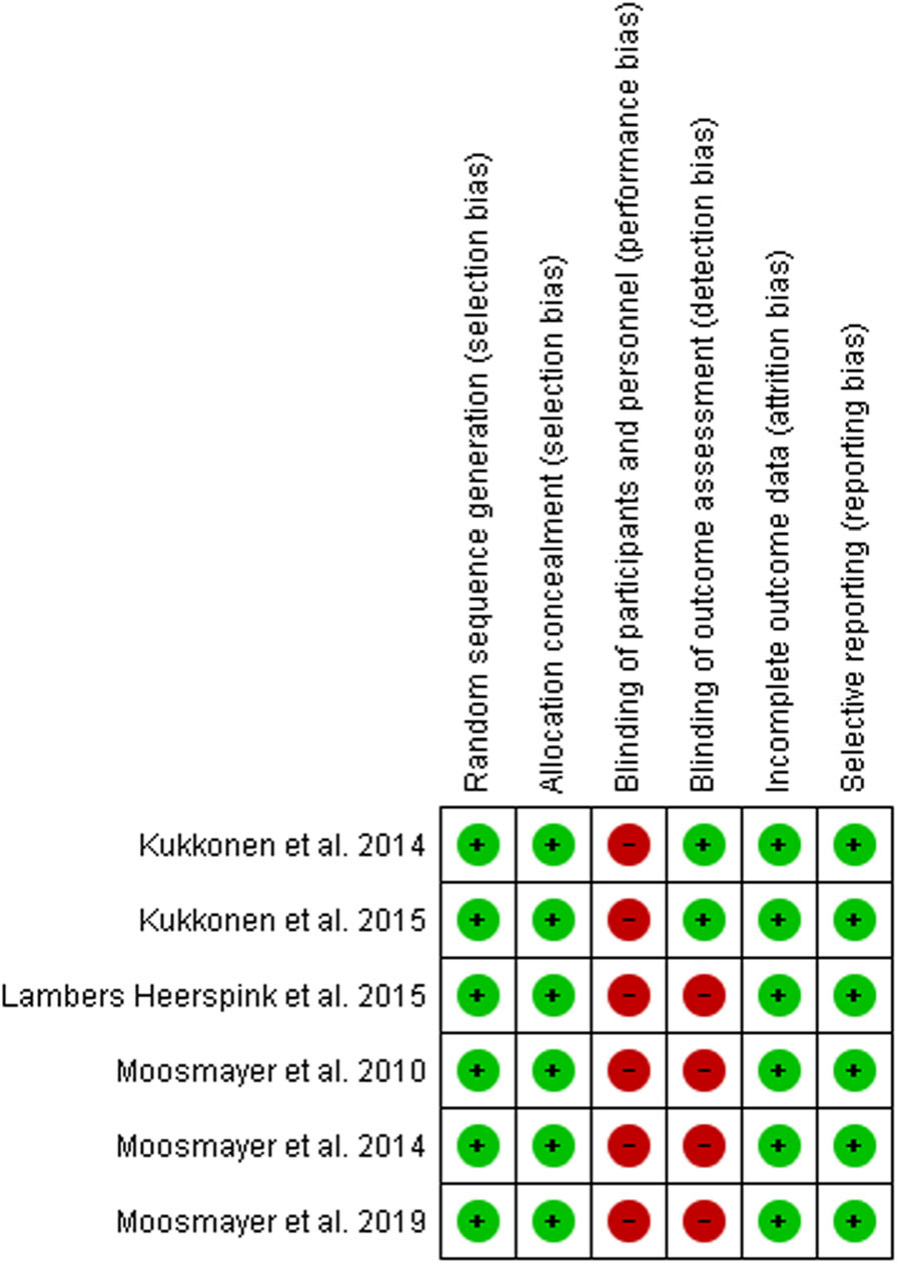
Risk of bias assessment

Please see the risk of bias summary presented in Fig. 5.

All the studies were judged as having a low risk of bias for selection bias because they reported the use of an appropriate method to generate the allocation schedule. Due to the lack of blinding of the patient and personnel, all the studies were judged as high risk for performance bias. Moreover, two out three patient’s cohorts were judge as having a high risk for the lack of blinding of outcome assessment. In contrast, Kukkonen et al. reported a low risk because the blinding of outcome assessors was ensured. However, we judge all the studies as having a low risk of bias for incomplete outcomes data since they reported less than 20% loss to follow-up and balanced loss among the groups. Other potential bias was not identified.

We assessed the quality of the results for each analysed variable (CMS at one-year follow-up, CMS at two years follow-up and VAS pain score at one-year follow-up). The quality of the evidence of the included studies was found to be high both for CMS at one year and two years of follow-up and for VAS pain score at one year of follow-up. We have presented the overall classification of the trials for these three main results in a single summary table of results (Table 5).

**Table 5:** GRADE: Summary of Findings

Outcomes	No. of participants (studies)	Certainty of the evidence (GRADE)
Constant-Murley score at 1-year of follow-up	257 (3 RCTs)	⨁⨁⨁⨁ HIGH
Constant-Murley score at 2-year of follow-up	211 (2 RCTs)	⨁⨁⨁⨁ HIGH
VAS pain score	147 (2 RCTs)	⨁⨁⨁⨁ HIGH

**GRADE Working Group grades of evidence**

**High certainty:** We are very confident that the true effect lies close to that of the estimate of the effect

**Moderate certainty:** We are moderately confident in the effect estimate: The true effect is likely to be close to the estimate of the effect, but there is a possibility that it is substantially different

**Low certainty:** Our confidence in the effect estimate is limited: The true effect may be substantially different from the estimate of the effect

**Very low certainty:** We have very little confidence in the effect estimate: The true effect is likely to be substantially different from the estimate of effect

## Discussions

RC tears are one of the most common disabling musculoskeletal disorders with high prevalence rate, and the appropriate treatment is still under debate [38, 39]. According to the American Academy Orthopaedic Surgeons (AAOS) guidelines, surgical RC repair is a valid option for patients with chronic, symptomatic full-thickness RC tears. However, the quality of evidence is unconvincing [40]. On the other hand, there is also a lack of supporting evidence for conservative treatment and, thus, the AAOS recommendations remain inconclusive [40]. Moreover, the superiority of surgical over the conservative treatment is challenging to demonstrate, due to heterogeneity of studies’ findings.

In the recent literature, three meta-analyses comparing the surgical and conservative treatment of RC tears are available, in which studies from up to June 2015, October 2016 and March 2018 were included [32–34]. Two of these compared surgical versus conservative management of full-thickness RC tears [32, 33], whereas the third added the evaluation of the subacromial decompression for the management of chronic/degenerative tears of the RC [34]. However, all the previous meta-analyses limited the comparison between surgical and conservative management at the short-term of follow-up (≤ 1 year). Moreover, they did not consider the percentage of retears in the surgical group.

In our meta-analysis, we performed the comparison between conservative and surgical management for patients with RC tears in terms of CMS (at 12 months and 24 months of follow-up) and VAS pain score (at 12 months of follow-up). One of the included randomised clinical trials reported results at 1, 2, 5 and 10 years of follow-up. The inclusion of this article allows us to perform the first comparison at two years of follow-up [14, 31]. Our meta-analysis provides the first comparison in the medium-term of follow-up in terms of CMS. These findings are similar to those measured in the short-term follow-up. In particular, no significant differences between the surgical group and the conservative group in terms of CMS were found (Fig. 2, Fig. 3). On the other hand, a better VAS pain was observed in favour of patients undergoing surgical repair at one year of follow-up (Fig. 4).

In the Norwegian study [31], at two years of follow-up, clinical outcomes were comparable for both surgical and conservative treatments; at five years of follow-up [31] both groups improved in term of clinical outcomes, but the CMS increased significantly in the surgical group; and at ten years of follow-up [13], clinical outcomes of patients undergoing surgical repair remained stable over time while the clinical outcomes of patients treated conservatively decreased, leading to the necessity of surgery in 14 of 51 patients (27%).

A hypothesis proposed to explain this phenomenon in the long-term is based on the inherent disadvantages of conservative treatment. Indeed, although the potential complications of surgical treatment (e.g., postoperative stiffness, infection) are not negligible, the conservative treatment does not restore the tendon, and this increases the risk of degeneration of shoulder tendons over time [41]. The strengths of this systematic review include the search strategy and the inclusion of only Level-1 studies. Nevertheless, there are several limitations. For instance, an extensive heterogeneity was found in the cohorts of patients analysed. Two studies enrolled patients with isolated supraspinatus tears [14, 30], one study enrolled patients with varying tears of RC (both infraspinatus, subscapularis and supraspinatus tears) [15]. In contrast, two did not specify the type of lesion [12, 31]. Moreover, due to the lack of information on the RC tear characteristics (e.g. tear size) in many studies, we were not able to conduct a subgroup analysis. The comparison of the type of intervention was challenging. One study added three corticosteroid injections to the standardised rehabilitation protocol in the conservative group [15]. Moreover, the number of sessions and the duration of physical therapy were not determined. Different surgical procedures were performed among the included studies: one cohort of patients was treated through arthroscopy [14, 30], whereas the other ones with open and mini-open approach [12, 15, 31]. Even though these techniques result to be equivalent, it is not clear whether the type of intervention may influence the functional outcomes and pain perception. Therefore, these results should be interpreted with caution. Besides, the comparison between muscle atrophy, adipose degeneration, size of the tear, and muscle retraction has not been reported in the included studies. This has prevented us from conducting a more rigorous quantitative analysis adjusted for these contextual factors. The long-term comparison of MRI findings is needed in future studies to investigate the potential impact of treatments on the progression of glenohumeral osteoarthritis, fatty infiltration, narrowing of the acromion-humeral distance and increasing of the size of the lesion.

## Conclusions

This is the first meta-analysis that compared surgical and conservative management for RC tears at two years of follow-up. The data reported in the included studies did not allow to draw a conclusion about muscle atrophy and the integrity of the repaired tendon. Further high-quality level-I randomised controlled trials at longer term follow-up are needed to evaluate whether surgical and conservative treatment provide comparable long-term results.

## Data Availability

The datasets used and/or analysed during the current study available from the corresponding author on reasonable request.

## Abbreviations

RC: Rotator Cuff
CMS: Constant-Murley score
PRISMA: Preferred Reporting Items for Systematic Reviews and Meta-Analyses
AAOS: American Academy Orthopaedic Surgeons
