# Correction to: Conservative versus surgical management for patients with rotator cuff tears: a systematic review and META-analysis

**DOI:** 10.1186/s12891-021-04525-w

**Published:** 2021-09-02

**Authors:** Umile Giuseppe Longo, Laura Risi Ambrogioni, Vincenzo Candela, Alessandra Berton, Arianna Carnevale, Emiliano Schena, Vincenzo Denaro

**Affiliations:** 1 Department of Orthopaedics and Trauma Surgery, Campus Bio-Medico University of Rome, Via Alvaro del Portillo, 200, 00128 Rome, Trigoria Italy; 2 Unit of Measurements and Biomedical Instrumentation, Campus Bio-Medico University of Rome, Via Alvaro del Portillo, 200, 00128 Rome, Trigoria Italy

**Correction to: BMC Musculoskelet Disord 22, 50 (2021)**

**https://doi.org/10.1186/s12891-020-03872-4**

Following publication of this article [1], the authors report the following Corrections to the main text:

i) The authors were made aware, that in the cohort of Kukkonen et al. [2, 3] the surgical group was labelled as conservative, and the conservative group as surgical. In view of this error, the authors corrected the database and performed the statistical analysis again (revised Table 3). The results were modified accordingly (revised Fig. 2, revised Fig. 3).
ii) The authors found that the main conclusion is unchanged, namely that there is no significant difference in terms of Constant and Murley score (CMS) between surgical and conservative treatment in patients with rotator cuff tears at two-year follow-up.
iii) Results showed statistically significant differences between the CMS measured at one year of follow-up, a secondary outcome, in favour of surgical rotator cuff repair compared with patients treated conservatively (*P* = 0.003).

**Table 3:** Constant and Murley Score (mean ± SD) at baseline, 12 and 24 months of follow-up

**Constant and Murley score at 1-year follow-up (range 0 to 100)**						
**Authors**	***Moosmayer 2010*, **[4]		***Kukkonen 2014*, **[3]		***Lambers Heerspink 2015*, **[5]	
	**Surgical group** **(n = 51)**	**Conservative group** **(n = 51)**	**Surgical group** **(n = 55)**	**Conservative group** **(n = 55)**	**Surgical group** **(n = 20)**	**Conservative group** **(n = 25)**
**Baseline**	35.3 ± 13.2	38.4 ± 14.2	58.1 ± 13.2	57.1 ± 16.7	55.6 ± 18.4	56.9 ± 15.0
**12 months**	77.7 ± 13.4	70.3 ± 19,1	77.9 ± 12.1	74.1 ± 14.2	81.9 ± 15.6	73.7 ± 18.4
**Constant and Murley score at 2-year follow-up (range 0 to 100)**						
**Authors**	***Moosmayer 2014*, **[6]		***Kukkonen 2015*, **[2]		-	
	**Surgical group** **(n = 51)**	**Conservative group** **(n = 51)**	**Surgical group** **(n = 54)**	**Conservative group** **(n = 55)**		
**24 months**	79.3 ± 13.6	77.7 ± 14.9	80.6 ± 15.4	76.2 ± 15.5

**Fig. 2:**
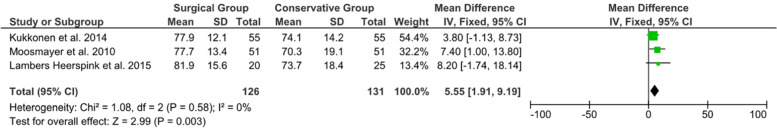
Forest plot: Constant and Murley score at 12 months of follow-up

**Fig. 3:**
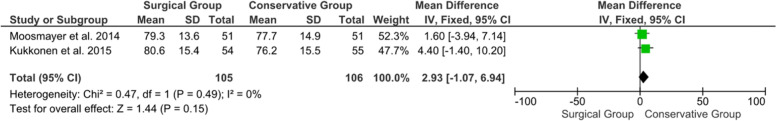
Forest plot: Constant and Murley score at 24 months of follow-up

The original article [1] has been updated.
